# A quantum computing approach to beam angle optimization

**Published:** 2025-04-10

**Authors:** Nimita Shinde, Ya-Nan Zhu, Haozheng Shen, Hao Gao

**Affiliations:** 1 Department of Radiation Oncology, University of Kansas Medical Center, USA; 2 University of Michigan-Shanghai Jiao Tong University Joint Institute, Shanghai Jiao Tong University, Shanghai, China

**Keywords:** beam angle optimization, mixed integer programming, quantum optimization

## Abstract

**Background:**

Beam angle optimization (BAO) is a critical component of radiation therapy (RT) treatment planning, where small changes in beam configuration can significantly impact treatment quality, especially for proton RT. Mathematically, BAO is a mixed integer programming (MIP) problem, which is NP-hard due to its exponential growing search space. Traditional optimization techniques often struggle with computational efficiency, necessitating the development of novel approaches.

**Purpose:**

This study introduces QC-BAO, a hybrid quantum-classical approach that leverages quantum computing to solve the MIP formulation of BAO.

**Methods:**

The proposed approach, QC-BAO, models BAO as an MIP problem, incorporating binary variables for beam angle selection and continuous variables for optimizing spot intensities for proton therapy. The proposed approach employs a hybrid quantum-classical framework, utilizing quantum computing to solve the binary decision component while integrating classical optimization techniques, including iterative convex relaxation and the alternating direction method of multipliers.

**Results:**

Computational experiments were conducted on clinical test cases to evaluate QC-BAO’s performance against clinically verified angles and a heuristic approach, GS-BAO. QC-BAO demonstrated improved treatment plan quality over both clinical and GS-BAO-selected angles. The method consistently increased the conformity index (CI) for target coverage while reducing mean and maximum doses to organs-at-risk (OAR). For instance, in the lung case, QC-BAO achieved a CI of 0.89, compared to 0.85 (clinical) and 0.76 (GS-BAO), while lowering the mean lung dose to 2.85 Gy from 3.36 Gy (clinical) and 4.80 Gy (GS-BAO). Additionally, QC-BAO produced the lowest objective function value, confirming its superior optimization capability.

**Conclusions:**

The findings highlight the potential of quantum computing to enhance the solution to BAO problem by demonstrated improvement in plan quality using the proposed method, QC-BAO. This study paves the way for future clinical implementation of quantum-accelerated optimization in RT.

## 1. Introduction

Radiation therapy (RT) plays a critical role in cancer treatment by delivering targeted radiation doses using multiple beam angles to tumors while minimizing exposure to healthy tissues and organs-at-risk (OAR). Beam angle optimization (BAO) [1] is a key aspect of RT treatment planning, selecting the optimal set of beam angles to achieve the best possible dose distribution. The selection of beam angles significantly impacts treatment plan quality in both intensity-modulated radiation therapy (IMRT) [2] and intensity-modulated proton therapy (IMPT) [3], as these plans often consist of only a few beam angles, and small changes in configuration can lead to notable differences in treatment outcomes [4].

Mathematically, BAO is a mixed-integer programming (MIP) [5,6] problem involving binary (0 or 1) variables for beam angle selection and continuous variables for optimizing spot intensities. However, BAO is a challenging combinatorial problem, with the number of possible beam angle combinations growing exponentially, leading to increasing computational complexity. Thus, BAO is classified as an NP-hard problem [7] and solving MIP formulation of BAO is computationally demanding due to its high-dimensional search space and complex constraints. Classical optimization methods, such as branch-and-bound algorithms [8], cutting-plane methods [9], and decomposition techniques [10], often struggle with efficiency.

To address these challenges, various methods have been proposed to tackle BAO, including heuristic and metaheuristic approaches [1,11–19], as well as group sparsity regularization techniques [20–23]. While these methods offer computational efficiency, they often yield suboptimal solutions. Thus, there is a need for improved approaches to achieve near-optimal beam angle selection efficiently.

Quantum computing (QC) [24,25] presents a promising alternative to solve MIP. By leveraging quantum parallelism, QC can explore multiple solutions simultaneously, offering potential speedups for combinatorial optimization problems. Variational quantum algorithms [26] and gate-based quantum algorithms efficiently solve binary optimization problems, making them well-suited for BAO. Recent advancements in QC have shown promise in radiation therapy optimization (see Section 4), and the increasing availability of quantum hardware, such as D-Wave’s quantum annealers [27] and IBM’s quantum processors [28], further supports its application in RT. To the best of our knowledge, quantum optimization has not yet been applied to solve the BAO problem.

This work introduces a novel quantum-classical hybrid approach, QC-BAO, to solve BAO by formulating it as an MIP and leveraging quantum optimization techniques. The proposed method systematically models the BAO problem and employs quantum computing to efficiently solve its binary component. A hybrid approach [26], integrating classical optimization methods with quantum algorithms, is used to identify near-optimal beam angle combinations. Computational experiments on clinical test cases demonstrate the effectiveness of this approach, indicating its potential for clinical implementation.

## 2. Methods

### 2.1. MIP model for beam angle optimization

For the problem of optimally selecting N angles from a set of B available beam angles, the proposed MIP optimization problem is

(1) $$\begin{array}{l} \underset{x,y}{min}\;f\left(d\right)\\ s.t.\;d=\sum_{i\in B}y_{i}\left(A^{i}x^{i}\right)\\ \;\;\;x^{i}\;\in \;\left\{0\right\}\;\cup \;[G,\;+\infty \}\;\forall i\in B\\ \;\;\;\sum_{i\in B}y_{i}=N\\ \;\;\;y_{i}\;\in \;\left\{0,1\right\}\;\forall i\in B. \end{array}$$

In Eq. (1), $A_{i}$ is the dose influence matrix for each beam angle i∈B, and G denotes the minimum monitor unit (MMU) threshold value. The continuous decision variable $x^{i}$ corresponds to the spot intensity vector for each beam angle i=1,…,B to be optimized, and the binary decision variable $y_{i}$ determines the beam angles selected in the plan. The first constraint in Eq. (1) defines the dose distribution d based on the selected beam angles $y_{i}$ and their corresponding spot intensity vectors $x^{i}$. The second constraint enforces the MMU requirement [29,30], ensuring the treatment plan deliverability. The third and fourth constraints together impose a binary restriction on $y_{i}$ and enforce the selection of exactly N beam angles from the available B angles.

The first term in the objective function, $f\left(d\right)$, is defined as

$$f\left(d\right)=\sum_{i=1}^{N_{1}}\frac{{\text{w}}_{\text{1}}}{n_{i}}{\left\|d_{\Omega_{1i}}-b_{1i}\right\|}_{2}^{2}\;+\;\sum_{i=1}^{N_{2}}\frac{{\text{w}}_{2}}{n_{i}}{\left\|d_{\Omega_{2i}}-b_{2i}\right\|}_{2}^{2}+\frac{{\text{w}}_{3}}{n_{i}}{\left\|d_{\Omega_{3}}-b_{3}\right\|}_{2}^{2}.$$

The three components of the objective function $f\left(d\right)$ are described below.

- **Target dose matching:** The first term represents $N_{1}$ least square error terms that measure the difference between the actual dose $d_{\Omega_{1i}}$ and the prescribed dose $b_{1i}$ for the target and OAR. Here, $\Omega_{1i}$ is the set of active indices, i.e., the set of voxel indices at which the actual dose differs from the prescribed dose value.
- **DVH-max constraint for OAR:** The second term in $f\left(d\right)$ incorporates $N_{2}$ dose volume histogram (DVH)-max constraints [31,32] for OAR. A DVH-max constraint ensures that at most a p fraction of the total voxels in a given OAR receive a dose exceeding $b_{2i}$. To enforce this, $\Omega_{2i}$ is defined as the set of indices violating the constraint. Let $d^{\prime }$ denote the dose d sorted in descending order and let $n_{i}$ be the number of voxels in OAR i. Then, $\Omega_{2i}=\left\{j|j\geq p\times n_{i}\right\}$ if $d_{p\times n_{i}}^{\prime }\geq b_{2i}$. Thus, if the DVH-max constraint is violated, the second term in $f\left(d\right)$ minimizes the least square error between the dose $d_{\Omega_{2i}}$, and the DVH-max dose $b_{2i}$.
- **DVH-min constraint for the target:** The third term enforces a DVH-min constraint [31,32] for the target, ensuring that at least a fraction p of the total target voxels receive a dose greater than $b_{3}$. Again, sorting the dose d in descending order as $d^{\prime }$ and letting n be the total number of voxels in the target, the active index set, $\Omega_{3}$, for DVH-min constraint is defined as $\Omega_{3}=\left\{j|j\leq p\times n\right\}$ if $d_{p\times n}^{\prime }\geq b_{3}$. If the DVH-min constraint is violated, the third term in $f\left(d\right)$ minimizes the least square error between the actual dose $d_{\Omega_{3}}$ at the violating indices and the minimum required dose $b_{3}$.

The solution to Eq. (1) is (i) a binary vector indicating the selected beam angles for dose delivery, and (ii) spot intensities corresponding to the selected beam angles. Section 2.2 presents an optimization method to solve the MIP formulation, Eq. (1), of the beam angle optimization problem.

### 2.2. QC-BAO method for beam angle optimization

The proposed QC-BAO method to solve the beam angle optimization problem follows a two-step process:

1. Step 1: Beam angle selection and spot intensity optimization:
  In the first step, Eq. (1) is solved to determine the optimal beam angle selection. A quantum optimization algorithm is employed to optimize the binary variables $y_{i}$, which represent the selection of beam angles. The details of the algorithm used to solve Eq. (1) are provided in Sections 2.3 and 2.4. This step also yields the corresponding spot intensities for the selected beam angles. However, since the primary focus here is on beam angle selection, there remains an opportunity to further refine the spot intensities.
2. Step 2: Additional spot intensity refinement:
  In the second step, Eq. (1) is re-solved with the beam angles fixed according to the $y_{i}$ ’s determined in Step 1. This reduces the problem into a continuous optimization problem focused solely on refining the spot intensities. By isolating the continuous variables, this step enables a more precise adjustment of dose distribution for the selected beam angles.

### 2.3. Solution methodology for QC-BAO method

#### Step 1 of QC-BAO:

The first step of the QC-BAO method involves solving Eq. (1). However, Eq. (1) contains non-convex constraints, making it difficult to solve directly using standard optimization techniques. To enable more tractable optimization, auxiliary variables are introduced, transforming the problem into a more structured form. This leads to the following reformulated problem

(2) $$\begin{array}{l} \underset{x,y,z}{min}\;f\left(d\right)\\ s.t.\;d=\sum_{i\in B}y_{i}\left(A^{i}x^{i}\right)\\ \begin{array}{l} \;\;\;x^{i}\;=z^{i}\;\forall i\in B\\ \;\;\;z^{i}\;\in \;\left\{0\right\}\;\cup \;[G,\;+\infty \}\;\forall i\in B \end{array}\\ \;\;\;\sum_{i\in B}y_{i}=N\\ \;\;\;y_{i}\;\in \;\left\{0,1\right\}\;\forall i\in B. \end{array}$$

Next, the augmented Lagrangian of Eq. (2) is defined as

(3) $$\begin{array}{l} \underset{x}{min}\;f\left(\sum_{i\in B}y_{i}\left(A^{i}x^{i}\right)\right)+\frac{\mu_{1}}{2}\sum_{i\in B}{\left\|x^{i}-z^{i}+\lambda_{1i}\right\|}_{2}^{2}+\frac{\mu_{2}}{2}{\left(\sum_{i\in B}y_{i}-N+\lambda_{2}\right)}^{2}\\ s.t.\;\;\;z^{i}\;\in \;\left\{0\right\}\;\cup \;[G,\;+\infty \}\;\forall i\in B\\ \;\;\;\;\;\;\;\;\;y_{i}\;\in \;\left\{0,1\right\}\;\forall i\in B. \end{array}$$

Eq. (3) is solved using iterative convex relaxation (ICR) [33,34] and alternating direction method of multipliers (ADMM) [35,36], both of which have been successfully applied to inverse optimization problems [37–45]. The iterative method updates the active index sets for the DVH constraints. This is followed by sequential updates of each decision variable while keeping the others fixed.

In this work, a quantum optimization algorithm [24,25] is employed to solve the binary optimization problem for updating $y_{i}$ in each iteration. An outline of the optimization method for solving Eq. (3) is given in Algorithm 1. Note that, the use of quantum algorithms for updating binary decision variables, together with the use of classical optimization algorithms in the rest of the optimization process results in a quantum-classical hybrid algorithm [26,46]. A detailed explanation of the process that updates the decision variables (including the binary decision variable using quantum algorithm) is provided in Section 2.4. The output of Algorithm 1 consists of the spot intensity vector $x^{i}$ and the binary variable $y_{i}$. In the first step, approximately 10 iterations of Algorithm 1 are performed to obtain the value of $y_{i}$.

$\bar{\underline{\begin{array}{l} \underline{\mathrm{Algorithm}\;1:\;\mathrm{Optimization}\;\mathrm{method}\;\mathrm{for}\;\mathrm{solving}\;\mathrm{Eq}.\;\left(3\right)\;}\\ \;\;\;\;\;\;1.\;\mathrm{Input}:\;\text{Choose}\;\text{parameters}\;{\text{μ}}_{\text{1}},{\text{μ}}_{2},w_{1},w_{2},w_{3}\\ \;\;\;\;\;\;2.\;\mathrm{Initialization}:\;\text{Randomly}\;\text{initialize}\;x,y.\;\text{Choose}\;\text{number}\;\text{of}\;\text{iterations}\;T.\\ \;\;\;\;\;\;3.\;\text{Set}\;z^{i}=x^{i},\;\lambda_{1i}=\lambda_{2}=0.\\ \;\;\;\;\;\;4.\;\text{For}\;t\text{=1,}\ldots \text{,}T\\ \;\;\;\;\;\;\;\;\;\;\;\;\;\;\;\text{a}.\;\mathrm{Identify}\;\mathrm{active}\;\mathrm{index}\;\mathrm{sets}:\;\text{Determine}\;\text{the}\;\text{active}\;\text{index}\;\text{sets}\;\Omega_{1i},\;\Omega_{2i},\;\Omega_{3}\;\text{for}\;\text{the}\;\text{DVH}\;\text{constraints,}\;\text{as}\;\text{outlined}\;\text{in}\;\text{Section}\;2.1.\\ \;\;\;\;\;\;\;\;\;\;\;\;\;\;\;\text{b}.\;\mathrm{Update}\;\mathrm{primal}\;\mathrm{variables}:\;\text{Sequentially}\;\text{update}\;\text{the}\;\text{primal}\;\text{variables}\;x^{i},z^{i},y_{i}\;\text{by}\;\text{fixing}\;\text{all}\;\text{other}\;\text{variables}\;\text{and}\;\text{solving}\;\text{the}\;\text{resulting}\;\text{minimization}\;\text{problem}\;\text{for}\;\text{each}.\\ \;\;\;\;\;\;\;\;\;\;\;\;\;\;\;\text{c}.\;\mathrm{Update}\;\mathrm{dual}\;\mathrm{variables}:\;\text{Perform}\;\text{the}\;\text{following}\;\text{updates:}\\ \;\;\;\;\;\;\;\;\;\;\;\;\;\;\;\;\;\;\;\;\;\;\;\;\;\;\;\;\;\;\;\;\;\;\;\;\;\;\;\;\;\;\;\;\;\;\;\;\;\;\;\;\;\;\;\;\;\;\;\;\;\;\;\;\;\;\;\;\lambda_{1i}=\lambda_{1i}+z^{i}-x^{i}\\ \;\;\;\;\;\;\;\;\;\;\;\;\;\;\;\;\;\;\;\;\;\;\;\;\;\;\;\;\;\;\;\;\;\;\;\;\;\;\;\;\;\;\;\;\;\;\;\;\;\;\;\;\;\;\;\;\;\;\;\;\;\;\;\;\;\;\;\;\lambda_{2}=\lambda_{2}+\sum_{i\in B}y_{i}-N.\\ \;\;\;\;\;\;\text{5}.\;\mathrm{Output}:\;x,y \end{array}}}$

#### Step 2 of QC-BAO:

Once a near-optimal set of beam angles is determined in the first step, Eq. (3) is solved again with $y_{i}$ fixed. The objective of this step is to further optimize the spot intensities for the selected beam angles. The solution methodology is similar to that outlined in Algorithm 1, however, in this step, $y_{i}$ remains fixed.

### 2.4. Updating primal variables in Algorithm 1

A detailed explanation of Step 4b of Algorithm 1 is provided in this subsection.

1. Updating $x^{i}$: For each i=1,…,B, fix all variables except $x^{i}$ in Eq. (3). Since the resulting minimization problem is unconstrained in $x^{i}$, solving it involves taking the first-order derivative of the objective function with respect to $x^{i}$ and setting it to zero. The value of $x^{i}$ is then obtained by solving the resulting linear system of equations.
2. Updating $z^{i}$: For each i=1,…,B, fix all variables except $z^{i}$ in Eq. (3). The resulting minimization problem has a closed form solution, which is determined using soft thresholding:
$$z^{i}=\left\{\begin{array}{ll} max\left(G,x^{i}-\lambda_{1i}\right), & if\;x^{i}-\lambda_{1i}\geq G\text{/}2\\ \;\;\;\;\;\;\;\;\;\;\;0, & otherwise. \end{array}\right.$$
3. Updating $y_{i}$: In Eq. (3), fix all variables except $y_{i}$. This results in a quadratic unconstrained binary optimization (QUBO) [47] problem. The QUBO formulation is well-studied in quantum computing literature. In this work, the QUBO problem is solved using a MATLAB solver ‘qubo’ to find the optimal value $y_{i}$ ’s at each iteration.

### 2.5. Materials

The effectiveness of the proposed QC-BAO method is demonstrated by comparing its performance against two benchmarks: clinically verified beam angles, and GS-BAO method [23], which employs group sparsity regularization for beam angle optimization. Three clinical test cases are considered: head-and-neck (HN) (8 Gy x 5 fractions), abdomen (6 Gy x 4 fractions), and lung case (2 Gy x 10 fractions).

For both the GS-BAO and QC-BAO methods, beam selection is performed from a set of 72 non-coplanar angles, comprising 24 equally spaced gantry angles for each of three couch angles (0°, 30°, and 60°) yielding N=72 in Eq. (1). The number of selected beam angles is fixed at B=4 for the HN case and B=3 for both the abdomen and lung cases. In the clinically verified plan, 0° couch angle is used and the gantry angles used are (45°, 135°, 225°, 315°) for the HN case and (0°, 120°, 240°) for the abdomen and lung cases. The dose influence matrix is generated using MatRad [48] with a spot width of 5 mm on a 3 mm³ dose grid. CTV-based planning is performed with clinically defined constraints for all test cases. All plans are normalized to ensure that 95% of the target region receives at least 100% of the prescribed dose.

Plan quality is assessed using the following metrics: (a) conformity index (CI), (b) maximum dose delivered to tumor $\left({\text{D}}_{\text{max}}\right)$, (c) mean and max doses delivered to OAR. CI is defined as $V_{100}^{2}/\left(V\times V_{100}^{\prime }\right)$, where $V_{100}$ is the target volume receiving at least 100% of the prescription dose, V is the total target volume, and $V_{100}^{\prime }$ is the total volume receiving at least 100% of the prescription dose. The normalized maximum dose ${\text{D}}_{\text{max}}$ is calculated as $\left({\text{D/D}}_{\text{p}}\right)\times 100\%$, where D is the maximum dose delivered to the tumor, and ${\text{D}}_{\text{p}}$ is the prescription dose.

## 3. Results

### 3.1 Selection of beam angles

In all three test cases, HN, abdomen, and lung, the QC-BAO method selects beam angles that are distinct from both the clinically approved angles and those generated by the GS-BAO method, highlighting its ability to explore alternative and potentially more effective configurations. In the HN case, QC-BAO employs two 0° couch angles and two 30° angles, with gantry angles distributed across 135°, 330°, 0°, and 90°, deviating from the symmetrical angles used clinically. For the abdomen case, QC-BAO introduces greater variation in couch angles (30°, 30°, 60°) compared to the uniform 0° in the clinical setup, and utilizes non-traditional gantry angles such as 180°, 285°, and 90°. In the lung case, while couch angles remain fixed at 0° (similar to the clinical configuration), the selected gantry angles (30°, 90°, 315°) differ substantially from both the clinical and GS-BAO plans, demonstrating enhanced directional flexibility.

### 3.2 Comparison of objective function value

Across all three test cases, QC-BAO consistently achieves a lower or comparable objective function value, reflecting its ability to deliver superior solution. In the HN case, QC-BAO yields an objective value of 3.72, outperforming both GS-BAO (3.94) and the clinical plan (4.42). For the abdomen case, QC-BAO matches the performance of GS-BAO approach with an objective value of 0.16, while still improving over the clinical plan’s value of 0.33. Most notably, in the lung case, QC-BAO achieves a significant reduction with a value of 7.00, compared to 12.53 for GS-BAO and 7.50 for the clinical plan. These improvements suggest that QC-BAO is more effective at balancing target coverage with OAR sparing, a result made possible through quantum-enhanced solution to MIP formulation of beam angle optimization problem.

### 3.3 Comparison of performance of QC-BAO with clinically verified angles and GS-BAO

In terms of target dose uniformity, the QC-BAO method consistently achieves better or equivalent results across all three cases. The conformity index (CI) is highest for QC-BAO in every test case: 0.66 for HN compared to 0.64 for GS-BAO and 0.62 for the clinical plan; 0.93 for the abdomen case, which is the highest among the three methods; and 0.89 for the lung case, improving over both GS-BAO (0.76) and the clinical plan (0.85). QC-BAO also improves performance in terms of the target ${\text{D}}_{\text{max}}$ value.

**Figure 1:**
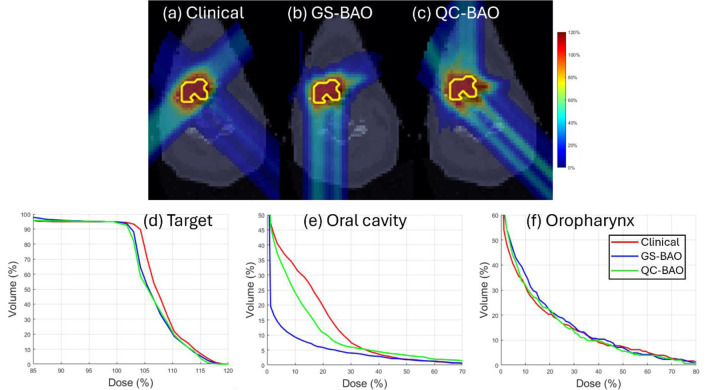
(HN02): (a)-(c) Dose plots, (d) DVH plot for target, (e)-(f) DVH plots for OAR

**Figure 2:**
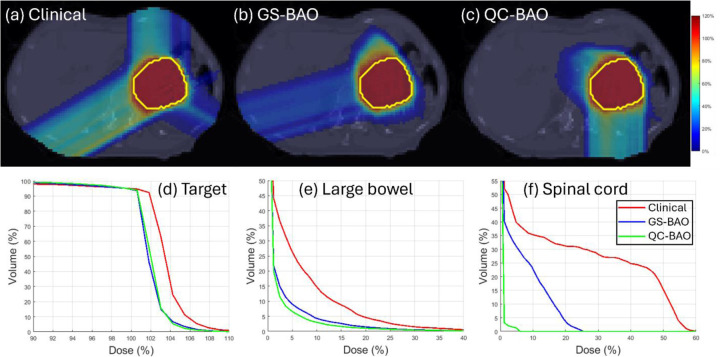
(Abdomen): (a)-(c) Dose plots, (d) DVH plot for target, (e)-(f) DVH plots for OAR

**Figure 3:**
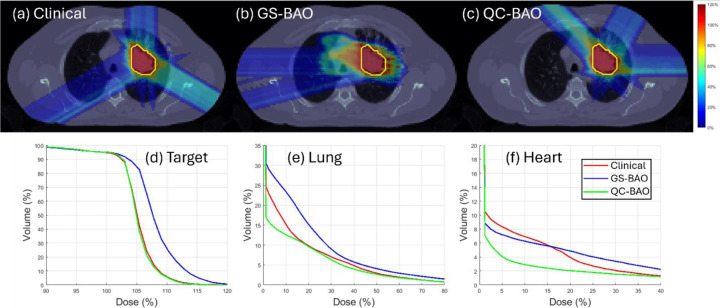
(Lung): (a)-(c) Dose plots, (d) DVH plot for target, (e)-(f) DVH plots for OAR

QC-BAO also demonstrates strong advantages in OAR sparing. In the HN case, the oral cavity ${\text{D}}_{\text{max}}$ is reduced to 37.12 Gy (lower than both GS-BAO and the clinical plan) while the ${\text{D}}_{\text{mean}}$ is effectively balanced at 3.03 Gy. For the oropharynx, QC-BAO delivers the lowest ${\text{D}}_{\text{max}}$ at 32.69 Gy. In the abdomen case, the QC-BAO method offers substantial dose reduction, particularly for the spinal cord, where the ${\text{D}}_{\text{max}}$ drops to 1.43 Gy compared to 6.05 Gy (GS-BAO) and 15.26 Gy (clinical). The spinal cord ${\text{D}}_{\text{mean}}$ is also minimized at just 0.03 Gy. In the lung case, QC-BAO continues to show superior sparing of critical OAR. It achieves the lowest lung ${\text{D}}_{\text{mean}}$ at 2.855 Gy and the lowest heart ${\text{D}}_{\text{mean}}$ at 0.795 Gy. These improvements are further illustrated by the DVH curves shown in Figures 1–3, which confirm the improved OAR sparing achieved by QC-BAO. Overall, the results highlight QC-BAO’s consistent ability to enhance target dose uniformity while substantially reducing OAR dose exposure.

## 4. Discussion

This work introduces a novel hybrid classical-quantum approach for solving the MIP formulation of BAO. The proposed method achieves superior optimization, yielding a lower objective function value compared to conventional techniques. Additionally, it reduces the dose to OAR, enhancing treatment quality. Computational experiments on clinical test cases validate its effectiveness, highlighting its potential for clinical implementation.

While the current approach significantly enhances computational efficiency, further refinements can improve performance. Developing problem-specific quantum algorithms tailored for BAO could optimize solution accuracy and further accelerate computation. Additionally, replacing the hybrid classical-quantum approach for solving the QUBO subproblem with a direct MIP solver [49] could further reduce computational overhead, increasing efficiency and scalability for real-world applications.

Beyond BAO, quantum computing holds promise for addressing other complex combinatorial optimization problems in RT that involve discrete decisions. In proton therapy, QC can improve energy layer optimization [50], leading to better dose distributions. Similarly, in spatially fractionated radiation therapy, QC can optimize LATTICE therapy peak placement [51] to enhance tumor control. These problems inherently involve binary decision-making, making them well-suited for QC-based MIP formulations.

Recent works have demonstrated the growing role of QC in RT applications. Quantum annealing has been explored for dose distribution optimization [52], significantly reducing computational time while maintaining treatment effectiveness. Additionally, quantum-enhanced machine learning models [53–55] have improved tumor segmentation and prediction, increasing accuracy and efficiency. These developments show the expanding role of QC in transforming radiation therapy optimization.

Further bolstering QC’s potential are advancements in quantum hardware, including improved qubit stability, error correction techniques, and increased qubit counts. Innovations in superconducting qubits (IBM [28], Google [56]), trapped ions (IonQ [57]), and quantum annealers (D-Wave [27]) have enhanced computational capabilities, making QC more practical for real-world applications. With more powerful quantum processors, RT optimization tasks that were previously infeasible due to computational complexity can now be tackled more efficiently, potentially enabling more precise and personalized treatment plans.

To integrate QC-enhanced BAO into clinical practice, rigorous validation is essential. Experimental validation [58,59] through phantom studies can provide quantitative performance metrics, paving the way for controlled clinical trials to ensure safety, efficacy, and practical implementation in patient treatments.

By integrating quantum computing into the BAO framework, this work addresses key computational challenges in RT treatment planning. The proposed approach improves the efficiency of beam angle selection, contributing to enhanced RT outcomes. Future research will focus on refining quantum algorithms, exploring direct MIP solutions using QC, and expanding QC applications to solve other critical problems in RT.

## 5. Conclusions

This work proposes a quantum optimization-based approach to solve mixed integer programming formulation of BAO to identify a near-optimal combination of beam angles from a set of available non-coplanar angles. The proposed model enhances both the objective function value of the MIP problem and the overall quality of the dose plan. Additionally, the integration of a quantum computing algorithm aids in efficiently solving the binary quadratic optimization problem.

## Figures and Tables

**Table 1:** Comparison of the output for the HN case. The best values in each row are highlighted in **bold**.

Structure	Quantity	Clinical	GS-BAO	QC-BAO
	Selected couch angles	(0°, 0°, 0°, 0°)	(30°, 30°, 30°, 60°)	(0°, 0°, 30°, 30°)
	Selected gantry angles	(45°, 135°, 225°, 315°)	(60°, 75°, 180°, 270°)	(135°, 330°, 0°, 90°)
CTV	Obj fn val	4.42	3.94	**3.72**
	${\text{D}}_{\text{max}}$	119.33%	118.40%	**117.82%**
	CI	0.62	0.64	**0.66**
Oral Cavity	${\text{D}}_{\text{max}}$	41.25 Gy	43.12 Gy	**37.12 Gy**
	${\text{D}}_{\text{mean}}$	3.62 Gy	**1.41 Gy**	3.03 Gy
Oropharynx	${\text{D}}_{\text{max}}$	35.79 Gy	34.17 Gy	**32.69 Gy**
	${\text{D}}_{\text{mean}}$	4.69 Gy	5.08 Gy	**4.69 Gy**

**Table 2:** Comparison of the output for the abdomen case. The best values in each row are highlighted in **bold**.

Structure	Quantity	Clinical	GS-BAO	QC-BAO
	Selected couch angles	(0°, 0°, 0°)	(0°, 60°, 60°)	(30°, 30°, 60°)
	Selected gantry angles	(0°, 120°, 240°)	(255°, 300°, 315°)	(180°, 285°, 90°)
CTV	Obj fn val	0.33	0.16	**0.16**
	${\text{D}}_{\text{max}}$	115.93%	**113.32%**	114.67%
	CI	0.89	0.92	**0.93**
L bowel	${\text{D}}_{\text{max}}$	20.38 Gy	**12.19 Gy**	15.61 Gy
	${\text{D}}_{\text{mean}}$	1.02 Gy	0.39 Gy	**0.30 Gy**
Spinal cord	${\text{D}}_{\text{max}}$	15.26 Gy	6.05 Gy	**1.43 Gy**
	${\text{D}}_{\text{mean}}$	3.84 Gy	1.09 Gy	**0.03 Gy**

**Table 3:** Comparison of the output for the lung case. V27 denotes percentage of OAR volume that receives at least 27 Gy dose. The best values in each row are highlighted in **bold**.

Structure	Quantity	Clinical	GS-BAO	QC-BAO
	Selected couch angles	(0°, 0°, 0°)	(0°, 0°, 30°)	(0°, 0°, 0°)
	Selected gantry angles	(0°, 120°, 240°)	(255°, 270°, 280°)	(30°, 90°, 315°)
CTV	Obj fn val	7.50	12.53	**7.00**
	${\text{D}}_{\text{max}}$	120.80%	126.03	**117.86%**
	CI	0.85	0.76	**0.89**
Lung	${\text{D}}_{\text{mean}}$	3.368 Gy	4.80 Gy	**2.855 Gy**
Heart	${\text{D}}_{\text{mean}}$	1.276 Gy	1.47 Gy	**0.795 Gy**
	V_27_	1.008%	1.71%	**0.972%**
